# Occurrence of typical antibiotics, representative antibiotic-resistant bacteria, and genes in fresh and stored source-separated human urine

**DOI:** 10.1016/j.envint.2020.106280

**Published:** 2021-01

**Authors:** Xiaoqin Zhou, Gabriela Jacqueline Perez Cuasquer, Zifu Li, Heinz Peter Mang, Yaping Lv

**Affiliations:** School of Energy and Environmental Engineering, Beijing Key Laboratory of Resource-oriented Treatment of Industrial Pollutants, University of Science and Technology Beijing, Beijing 100083, PR China

**Keywords:** Source-separated human urine, Antibiotics, Antibiotic-resistant bacteria, Antibiotic-resistant genes, Short-time storage, Elimination

## Abstract

Human urine is a source of fertilizer and, with proper management, it can be reused in agriculture. Determining the contamination issue of antibiotics in source-separated urine is important because the majority of antibiotics are excreted with urine. In this study, source-separated urine samples were randomly collected from a male toilet in a university building and analyzed in terms of 30 typical antibiotics (including 14 sulfonamides, 4 tetracyclines, and 12 fluoroquinolones) and tetracycline-resistant *Escherichia coli*, as well as its antibiotic-resistant genes to determine the contamination characteristics of antibiotic-related pollution in fresh and stored urine. Results showed that 18 out of 30 typical antibiotics were detected in fresh source-separated human urine. The dominant antibiotic was oxytetracycline with a frequency of 100%, followed by tetracycline, sparfloxacin, enrofloxacin, and ofloxacin, which demonstrated a detection frequency of 55%. Among the detected values, sulfonamides (2 antibiotics), tetracyclines (4 antibiotics), and fluoroquinolones (12 antibiotics) had a concentration range of 0.25–2.94, 0.94–41.2, and 0.06–163.16 ng/mL, respectively. Furthermore, tetracycline-resistant *Escherichia coli*, which was measured using plate count method, and its related gene, *tet* M, exhibited a maximum cell density of (200,000 ± 5000) CFU/100 mL and (2.73 ± 0.261) × 10^7^ copies/mL, respectively. When the fresh urine was stored in an ambient environment for 30 days to simulate the real circumstances of urine management, a significant reduction in antibiotics and antibiotic-resistant bacteria was observed, while the change in antibiotic-resistant genes was insignificant. The results of this study suggest that risks associated with antibiotics and their antibiotic-resistant bacteria and genes are retained during collection and storage. Hence, these kinds of microcontaminants must be considered in further urine utilization.

## Highlights

• 20 randomly real urine samples were collected and analyzed.
• Totally 30 antibiotics in the fresh real urine were identified.
• Representative antibiotic resistance bacteria and its gene were identified.
• Changes of antibiotics, antibiotic resistance bacteria and gens of urine were monitored.

## Graphical abstract

**Figure:**
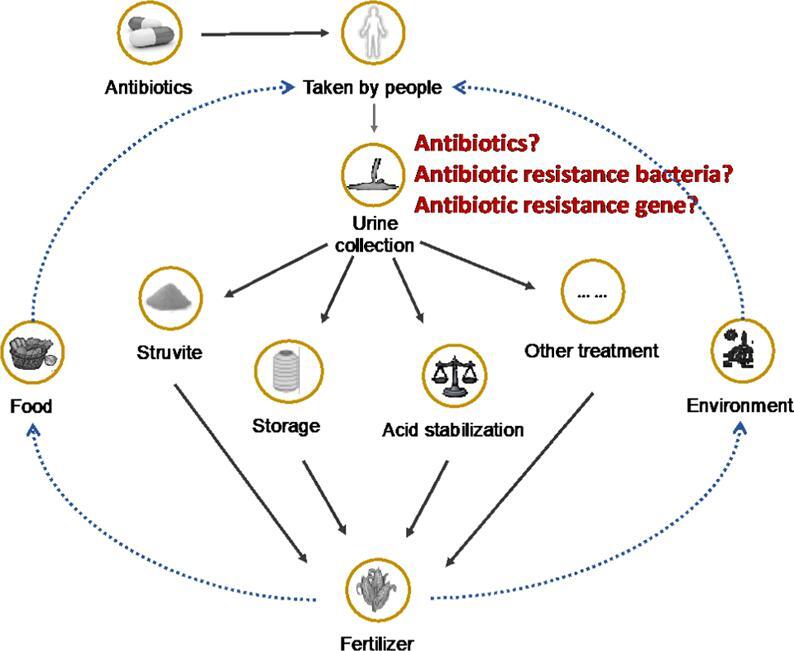

## 1 Introduction

Human urine may be collected separately in toilets by using sanitation technologies to achieve nutrient recovery because nitrogen, phosphorus, and potassium in urine represent nearly 80%, 50%, and 50% of necessary nutrients in agriculture, respectively (Bischel et al., 2015). Source separation of urine has been increasingly investigated and proposed as a method for efficient fertilizer production to make use of urine and avoid the discharge of large amounts of nutrients in surface water, which can cause eutrophication problems in rivers, lakes, and oceans (Udert et al., 2006). In addition to nutrients, urine also contains micropollutants, such as synthetic hormones and pharmaceuticals and their metabolites, including antibiotics excreted in the urine, which are extensively used by humans in the treatment of various diseases (Jia et al., 2012, Zhou et al., 2013). Antibiotics are only partially digested and absorbed by the human body, and approximately 30%–90% are excreted through urine and feces within 8–24 h after consumption (Frade et al., 2014). For example, the concentration of doxorubicin in a patient’s urine sample collected at 0–4 h ranges from 9.44 mg/L to 17.87 mg/L, which is nearly 95 times the amount of plasma in patients (Gao et al., 2020). Meanwhile, ciprofloxacin is reportedly excreted by 65% in the urine while only 25% is excreted in the feces; thus, urine contains most of excreted antibiotics (Frade et al., 2014). China is the largest producer of antibiotics worldwide with a yearly production of 210,000 tons (Zhang et al., 2015). China is also the largest consumer of antibiotics, and 90% of the produced antibiotics are applied in agriculture (48%) and medicine (42%) (Zhou et al., 2013). However, the indiscriminate use of antibiotics has generated a survival response in microorganisms; this survival response is called antibiotic resistance (Jia et al., 2012), which allows microorganisms to evade the bactericidal action of some agents efficiently and become emerging contaminants that can affect human health and the environmental ecosystem through the direct consumption of contaminated water and/or food (Lamba and Ahammad, 2017, Lind et al., 2001, Zhou et al., 2013). In the US, antibiotic-resistant bacteria and fungi caused at least 2,868,700 infections and 35,900 deaths annually in 2019 (U.S. Centers for Disease Control and Prevention, 2019). According to the World Health Organization, antibiotic resistance has become a critical global public health issue in this century (WHO, 2017). Currently, the majority of studies focus on antibiotic identification in wastewater, especially the fate of antibiotics during the wastewater treatment process, and the health risks associated with antibiotic resistance (Kraemer et al., 2019, Larsson, 2014). Wastewater treatment plants (WWTPs) have been considered reservoirs for antibiotic-resistant genes (Manaia et al., 2018). Human excreta accounts for only 1% of the volume of domestic wastewater but the greatest amount of pollutants. Thus, determining the trend of antibiotic contamination level in source-separated urine when urine is used as fertilizer is important. However, to the best of our knowledge, studies on such topics are limited.

Although source separation has been promoted since the 1990s, previous studies focused on nutrients in urine, and limited studies have been conducted in understanding antibiotics, antibiotic-resistant bacteria and genes. A case study on the behavior of pharmaceuticals related to the treatment of human immunodeficiency virus (HIV) investigated the liquid phase of source-separated urine during storage; however, artificial compounds rather than the real condition of human urine were used in the study (Jaatinen et al., 2016). A study in eThekwini, South Africa, investigated the municipality-scale production of agricultural fertilizers from human urine collected from urine-diverting dry toilets (Bischel et al., 2015). In this case, the presence of sulfamethoxazole was detected in 95% of samples, with a maximum concentration of 6800 ug/L. Recently, research in Lusaka, Zambia, demonstrated that sulfamethoxazole in source-separated urine had a concentration of 7740 µg/L, which was several orders of magnitude higher than that in wastewater (Ngumba et al., 2020). The application of fertilizer by excreta introduced antibiotic-resistant genes to crops and subsequently developed antibiotic resistance in the food chain from wastewater, thereby raising the issue of antibiotic resistance in organic fertilizer (Chen et al., 2017, European Commission DG Environment News Alert Service, 2018). Urine and feces from users may contain considerable amounts of active residues. A study found that antibiotics and antibiotic-resistant genes exist in manure-fertilized soil and a nearby watershed (Camotti Bastos et al., 2018). Thus, if urine is applied as fertilizer, then the occurrence and concentrations of antibiotics must be investigated for the safe usage of urine in agriculture.

On the basis of the above-mentioned points, this study aims to identify the presence of typical antibiotics in source-separated human urine and evaluate their contamination level and detection frequency to address the existing research gap. Tetracycline-resistant *Escherichia coli (E. coli)* was selected as representative antibiotic-resistant bacteria to determine the potential microbial risk caused by antibiotics. The urine was monitored with antibiotics, tetracycline-resistant *E. coli* and its genes change for 30 days to understand the behavior of antibiotics and its potential health effect during storage. The results can provide useful information for controlling potential health risks when source-separated urine is intended for agricultural use.

## 2 Materials and methods

### 2.1 Urine samples

Urine samples were collected from a male toilet in an office building of the University of Science and Technology Beijing. The building has 12 floors, and each floor has a male toilet with two urinals; the two urinals in the toilet of the 12th floor were transformed into waterless urinals. Thus, the male toilet of the 12th floor was selected as the sampling point for the collection of non-flushed source-separated urine. During the sample collection period, a sterile plastic barrel was directly connected with the urinal at the bottom. The toilet users are mainly young researchers, including master’s and PhD candidates, numbering a total of about 30 volunteers. The sampling was conducted during early summer in Beijing under ambient temperature from 8:00 a.m. to 11:00 a.m., and the collected volume was 3–5 L each time. Samples were collected once each day, and the total sampling time was 20 days; sampling was conducted randomly, not consecutively (May 21st to June 13th, 2019). All the urine samples were transported to the laboratory and immediately prepared for further analysis once sampling was finished.

The sampling conditions of each time are listed in Table 1. The average temperature of source-separated urine in 20 sampling days was (26.2 ± 1.5) °C, which ranged from 22.7 °C to 28.9 °C, and the mean pH was (9.11 ± 0.046). The range of ammonia during sampling was 4593.2–6627.2 mg/L NH_4_–N with an average of 5158 mg/L NH_4_–N. Urea in the fresh urine is easily hydrolyzed into ammonia, resulting in increased pH and high ammonia concentration. Hence, the collected urine samples were observed immediately after hydrolysis.

**Table 1:** Basic chemical parameters of collected urine samples.

N° Samples	Temperature (°C)	pH	Ammonia, mg/L NH_4_- N
1	22.7	9.15	4631.6
2	26	9.09	6254.9
3	25.4	9.12	6100.0
4	25.8	9.15	6031.5
5	26.8	9.11	6627.2
6	25.9	9.14	6143.2
7	24.6	9.14	6031.5
8	25.7	9.12	4593.2
9	25.6	9.12	4657.0
10	23.9	9.11	4650.0
11	25.7	9.08	4695.3
12	26.2	9.03	4600.0
13	26.4	8.97	4784.6
14	26.6	9.17	4720.8
15	27.7	9.13	4657.6
16	28.9	9.13	4784.6
17	26.6	9.13	4800.0
18	26.7	9.13	4790.0
19	28.1	9.16	4810.0
20	28.9	9.11	4799.0

In practice, urine-diverting toilets collect urine over time. The collected urine is then stored for weeks or months for further treatment or utilization. Hence, the change in the substrate concentrations of urine sample was investigated. Urine samples were collected three times within three days from the same toilet mentioned above. Thereafter, the urine samples were mixed to make 6 L and distributed into three glass bottles as parallels for 30 days of storage under ambient temperature of 27.1 °C ± 0.8 °C. The urine samples were divided into three bottles to avoid cross contamination during sampling. Table 2 presents the pH and ammonia concentrations of the fresh samples and after mixture, and results showed that the urine samples hydrolyzed after mixing. Samples were taken every 5 days to analyze the pH, ammonia, antibiotics, and antibiotic-resistant bacteria and genes. In addition, tetracycline-resistant *E. coli* was analyzed every 6 h during the first 36 h of storage due to rapid elimination.

**Table 2:** pH and ammonia concentration before and after mixture.

N° Samples	pH	Ammonia, mg/L NH_4_- N
Sample 1	8.57	1693
Sample 2	8.49	2907.2
Sample 3	8.55	1953.7
Mixture	9.1	4631.6

### 2.2 Chemical and microbial analysis

Table 3 lists 30 typical antibiotics for the analysis of source-separated urine; these antibiotics are frequently used in China and detected in wastewater surface water, rivers, and sediments (Lu et al., 2018, Peng et al., 2011, Qiao et al., 2018, Zhang et al., 2015, Zhou et al., 2013). The chemical analysis of the antibiotics proceeded with modifications; refer to the literature for details (Yin et al., 2018). Liquid chromatography–mass spectrometry was used to analyze the extracted antibiotics; the device is an Agilent 1200 Series HPLC system (Agilent, Palo Alto, CA, USA) coupled to Applied Biosystems API4000 triple quadrupole mass spectrometer with electrospray ionization interface (Foster City, CA, USA) in positive mode. Nitrogen gas (99.9% purity) was used as the drying and collision gas. Agilent Eclipse XDB-C18 (150 mm × 2.1 mm, 3.5 μm) analytical column (Agilent Technologies, Atlanta, GA) operated at 35 °C was used for chromatographic separation, and the injection volume of the samples was 5 μL. A total of 0.1% formic acid in water (mobile phase A) with 0.1% formic acid in methonal (mobile phase B) were used as mobile phase gradient, which was run at a flow rate of 0.3 mL min^−1^, and the operation condition was 95% A for 0–1 min, 95%–65% A for 1–2 min, 65–50% A for 2–4.5 min, 50%–35% A for 4.5–6 min, 35% A for 6–8 min, 35%–5% A for 8–10 min, 5% A for 10–12 min, and 5%–95% A for 12–12.1 min, then continued for 4 min. Mass spectrometer was adjusted by using an optimizer (Agilent, Palo Alto, CA, USA) in terms of decluttering potential, collision energy, and multiple reaction monitoring mode independently. The operation condition of the ion source was an ion spray voltage of 5000 V; ion source at a temperature of 500 °C; curtain gas at 30 psi; atomization air pressure of 60 psi; auxiliary gas of 60 psi; entrance potential at an voltage of 10 V; collision cell exit potential at a voltage of 12 V; and dwell time of 30 ms for each transition. Analyte 1.5.2 (Applied Biosystems) was used for data acquisition and instrument control. More details on standards and stock solution, reagents and solvents, and sample preparation are listed in the Supplementary Information.

**Table 3:** Identified antibiotics in source-separated urine.

Sulfonamides	Tetracyclines	Fluoroquinolones
Sulfadiazine	Tetracycline	Norfloxacin
Sulfadimethylpyrimidine	Oxytetracycline	Fleroxacin
Sulfachlorpyridazine	Chlortetracycline/	Sparfloxacin
Sulfamethoxazole	Doxycycline	Orbifloxacin
Sulfamethoxine		Enrofloxacin
Sulfathiazole		Pefloxacin
Sulfamethazine		Difloxacin
Sulfaguanidine		Ciprofloxacin
Sulfamonomethoxine		Sarafloxacin
Sulfaphenazole		Lomefloxacin
Sodium sulfacetamide		Ofloxacin
Oxolinic acid		Danofloxacin
Sulfametoxydiazine		
Sulfamethoxypyridiazine		

To detect tetracycline-resistant *E. coli* from the urine samples, a certain volume of urine sample was extracted by using a sterile filter membrane (0.45 μm). The resistant bacteria were selectively separated by placing tetracycline with concentrations of 4, 16, and 30 µg/ml in Endo agar and then by culturing at 37 °C for 24 h. Thereafter, the membrane was transferred to NA-MUG agar medium in 37 °C for another 4 h of incubation. Subsequently, *E. coli* was counted under ultraviolet light (6 W at 366 nm) with colony edge or blue fluorescence generation. Antibiotic-resistant genes (ARGs) were identified from the presence of *tet* A, *tet* Q, and *tet* M via PCR. The reaction system is specified in Table 4 (Chen and Zhang, 2013). Data analysis was performed using Origin 8.0.

**Table 4:** Primer sequences used for PCR in antibiotic-resistant genes.

N	Primer name	Primer sequence	Product length/bp	Tm/°C
1	Tet A-F tet A-R	GCTACATCCTGCTTGCCTTC CATAGATCGCCGTGAAGAGG	210	60
2	tet M-F tet M-R	ACAGAAAGCTTATTATATAAC TGGCGTGTCTATGATGTTCAC	171	55
3	tet Q-F tet Q-R	AGAATCTGCTGTTTGCCAGTG CGGAGTGTCAATGATATTGCA	169	63

## 3 Results and discussion

### 3.1 Occurrence of antibiotics in fresh source-separated urine

**Fig. 1:**
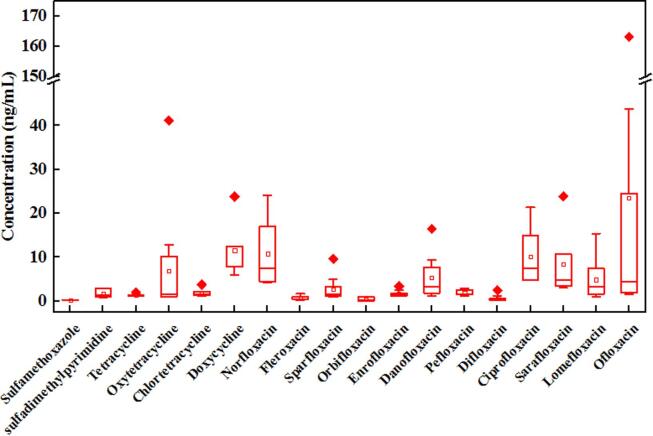
Box chart of the summary statistics of antibiotic concentration ranges of sulfonamides, tetracyclines, and fluoroquinolones detected in male source-separated urine in 20 sampling days. Note: Only some of the antibiotics described were detected in the collected samples. (Legend: boxes: 25%–75%, whiskers: 5%–95%, diamond: data, dots: maximum and minimum values, crosses: 99% and 1% values, and square: mean value).

This study detected 18 out of 30 target antibiotics, including 2 sulfonamides and 4 tetracyclines; the remainder included fluoroquinolones, with concentration ranges of 0.25–2.94, 0.94–23.79, and 0.06–163.16 ng/mL. Only sulfamethoxazole and sulfadimethylpyrimidine were identified in the samples from the 14 antibiotics listed in the sulfonamide group (Fig. 1). Sulfamethoxazole was detected in 45% of the samples (0.84–2.94 ng/mL), while sulfadimethylpyrimidine was detected in 2 out of the 20 samples with concentrations of 0.25 and 0.29 ng/mL. In the tetracycline group, oxytetracycline was detected in 100% of the samples with a concentration range of 0.94–41.2 ng/ml. Tetracycline, chlortetracycline, and doxycycline were detected in 55%, 35%, and 25% of the samples, with concentration ranges of 1.16–2.09, 1.2–3.76, and 5.98–23.79 ng/mL, respectively. Twelve antibiotics in the fluoroquinolone group were identified in the samples, with predominant compounds of ofloxacin, norfloxacin, sarafloxacin, ciprofloxacin, danofloxacin, and lomefloxacin, which were detected in concentration ranges of 1.60–163.16, 4.24–24.03, 3.02–23.90, 4.75–21.38, 1.13–16.45, and 1.05–15.42 ng/mL, respectively, indicating a frequency of 55%, 30%, 55%, 30%, 40%, 35%, and accordingly. Low quantities of fleroxacin, sparfloxacin, orbifloxacin, enrofloxacin, pefloxacin, and difloxacin were detected with concentration ranges of 0.24–1.71, 0.99–9.66, 0.06–1, 1.13–3.41, 1.27–2.88, and 0.22–2.51 ng/mL, respectively. Obviously, the majority of antibiotics detected in this study belong to the fluoroquinolone group, which is also frequently detected at high concentrations in the environment, including wastewater, surface water, groundwater, and even drinking water (Jia et al., 2012, Lu et al., 2018, Peng et al., 2011).

Table 5 presents the antibiotic concentration findings in this study and those reported in different studies. Overall concentrations of detected antibiotics in urine samples were higher than the findings reported in other water samples, such as raw wastewater, treated wastewater and surface water. Sulfamethoxazole was detected in WWTPs with concentration ranges of 0.083–0.455 ng/mL (average of 0.303 ng/mL) in the influent and 0.037–0.316 ng/mL (average of 0.157 ng/mL) in the final effluent (Peng et al., 2011); these values are lower than 10% of the concentration values detected in this study. For the tetracycline group, a particular case study in Wisconsin detected oxytetracycline and chlortetracycline in effluents of WWTPs with concentrations of 4.2 and 0.42 ng/mL, respectively (Karthikeyan and Meyer, 2006), which were significantly lower than the value in the current findings. However, another case demonstrated that oxytetracycline is a frequently detected antibiotic in China with a maximum concentration of 72.9 ng/mL (Suzuki and Hoa, 2012). Doxycycline was detected in WWTP with a concentration of 6.7 ng/ml (Borghi et al., 2015), which is 70% less than our findings. For the fluoroquinolone group, norfloxacin was detected with a concentration range of 0.027–0.489 ng/mL in wastewater (Liu et al., 2012), which is approximately one-fiftieth of the finding in the current study. Enrofloxacin was detected in WWTP in Wisconsin with a concentration of 0.27 ng/mL (Karthikeyan and Meyer, 2006), which is even lower than 7% of the value in the current study. Ciprofloxacin was detected in hospital effluents at concentrations of 0.7 and 124.5 ng/mL (Kummerer, 2003), which is six times higher than those in the current study. Meanwhile, ciprofloxacin was detected with concentration ranges of 60–90 and 20–80 ng/L in two typical WWTPs in south China, respectively (Zhou et al., 2013). These values are much lower than the concentration detected in this study. Indian cities registered a concentration range of 0.212–160 ng/mL (Singh et al., 2019), which is similar to the findings in the current study. Notably, the urine samples used in this study were undiluted, being 100 times concentrated compared with the urine that enters wastewater through a large amount of flushing water and graywater, even with rainwater in some cases. From Table 5, we can conclude that the antibiotics in source-separated urine are overall 10 times higher than those in raw wastewater, indicating that source separation can not only concentrate on nutrients at the source but can also be beneficial for antibiotic removal. Besides, the concentrations of the detected antibiotic in the urine samples were even higher than the value reported in rivers in China. For example, the maximum concentration of sulfamethoxazole in Ravi River was 2700 ng/L, and Norfloxacin was reported with concentrations of 6800 ng/L and 1100 ng/L in Hai River system and Pearl River (Singh et al., 2019).

**Table 5:** Comparison of antibiotic concentrations between the current study and other water samples.

	Antibiotic	Range (ng/mL)	Other water samples(ng/mL)	References
Sulfonamide	Sulfadimethylpyrimidine	0.25–0.29	NR	
	Sulfamethoxazole	0.84–2.94	0.083–0.455 0.037–0.316	Peng et al., 2011
Tetracyclines	Tetracycline	1.16–2.09	0.0103	Suzuki and Hoa, 2012
	Oxytetracycline	0.94–41.2	4.2,72.9	Karthikeyan and Meyer, 2006, Suzuki and Hoa, 2012
	Chlortetracycline	1.2–3.76	0.42	
	Doxycycline	5.98–23.79	6.7	Borghi et al., 2015
Fluoroquinolones	Norfloxacin	4.24–24.03	0.027–0.489	Liu et al., 2012
	Fleroxacin	0.24–1.71	0.014	Jia et al.,2012
	Sparfloxacin	0.99–9.66	0.004	Jia et al.,2012
	Orbifloxacin	0.06–1	NR	
	Enrofloxacin	1.13–3.41	0.27	Karthikeyan and Meyer, 2006
	Danofloxacin	1.13–16.45	0.07	Borghi et al., 2015
	Pefloxacin	1.27–2.88	NR	
	Difloxacin	0.22–2.51	NR	
	Ciprofloxacin	4.75–21.38	0.7–160	Kummerer, 2003, Zhou et al., 2013, Singh et al., 2019
	Sarafloxacin	3.02–23.9	3.02–23.9	Kummerer, 2003
	Lomefloxacin	1.05–15.42	0.162	Jia et al.,2012
	Ofloxacin	1.6–163.16	0.212–160	Singh et al., 2019

NR: No reference.

Moreover, the concertation of antibiotics in this study was lower than the reported value in other countries. As mentioned previously, sulfamethoxazole was detected in source-separated urine in eThekwini, South Africa, with high concentrations of approximately 6800 ng/ml (Bischel et al., 2015), which is 2312 times greater than the findings in the current study mainly because sulfamethoxazole is commonly used by HIV-positive patients. Thus, comparing this value with the data in this study is unnecessary. Moreover, the concentration of sulfamethoxazole in source-separated urine sampled in Lusaka, Zambia, was 7740 µg/L, which is thousands of orders of magnitude higher than that in this study (Ngumba et al., 2020). On the basis of the above-mentioned comparisons, antibiotic pollution varies considerably in terms of species and concentrations, which is highly dependent on the individual consumption of antibiotics; more attention should be paid to developing countries and rural regions in this regard. In this study, the fluoroquinolone group was detected with high frequency, suggesting that fluoroquinolones were commonly used among the toilet users. The urine samples collected in this study were mainly from young people. Theoretically, these individuals have strong immune systems; thus, the concentration of such antibiotics should be potentially low.

Notably, sarafloxacin was detected in the concentration range of 3.02–23.9 ng/mL in this study, but this antibiotic was approved only for the prevention of poultry diseases (Kummerer, 2003). Table 6 lists the antibiotics in the current study and their different usages in human and animal infections. To the best of our knowledge, rivers increase the susceptibility of humans and animals to illness because wastewater from hospitals and livestock feedlots as well as effluent and sewage sludge from municipal sewage treatment plants that end up in rivers contain antibiotics to some extent (Jia et al., 2012). Thus, antibiotics transformed within the whole circle, thereby persisting in the environment. Emphasizing the antibiotic contamination of source-separated urine may be an efficient way to control health risks, especially when urine is utilized as fertilizer for eatable crops.

**Table 6:** Usage of different antibiotics for humans and animals.

Antibiotics	antibiotic usages for anti-infections				References
	Human	Animal			
		Pig	Poultry	Cattle	
Sulfamethoxazole Sulfadimethylpyrimidine	√	√	√	√	Zhou et al., 2013
Norfloxacin, Fleroxacin	√	√	√		Suzuki and Hoa, 2012
Enrofloxacin			√		Zhang et al, 2015
Danofloxacin		√	√	√	Schneider et al,1993
Pefloxacin, Ciprofloxacin	√	√	√		Suzuki and Hoa, 2012
Sarafloxacin			√		Kummerer, 2003
Ofloxacin	√	√	√		Zhang et al., 2015, Singh et al., 2019
Danofloxacin		√	√	√	Kibret and Abera (2011)
Tetracycline, Oxytetracycline, Chlortetracycline, Doxycycline	√	√	√	√	Zhang et al, 2015,

### 3.2 Occurrence of antibiotic-resistant bacteria and genes in fresh source-separated urine

**Fig. 2:**
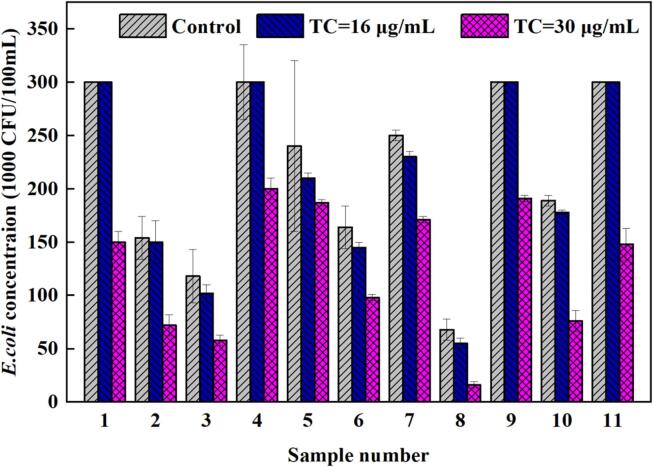
*E. coli* resistant at 16 and 30 µg/mL of tetracyclines.

*E. coli* was incubated at 4, 16, and 30 µg/mL of tetracyclines in the media to assess the potential concentration of antibiotic resistance in fresh urine. The culture media remained unchanged at a tetracycline dosage of 4 µg/mL and without antibiotic. Eleven out of 20 samples were tested with live *E. coli* at concentrations of 16 and 30 µg/mL (Fig. 2). Hence, the detection frequency of antibiotic-resistant bacteria was 55%. The range of density of colonies in control plates was 68,000–300,000 CFU/100 mL, and the number of colonies was approximately 55,000–300,000 and 16,000–200,000 CFU/100 mL with 16 and 30 µg/mL of tetracyclines, respectively.

**Fig. 3:**
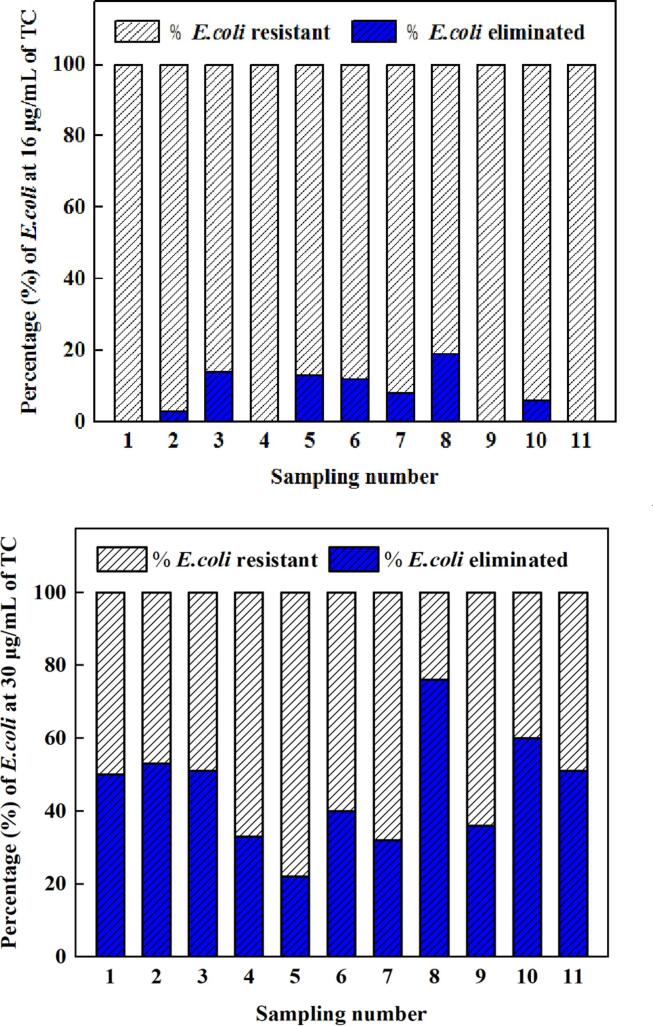
Percentage of *E. coli* eliminated at (a) 16 and (b) 30 µg/mL of tetracyclines.

The elimination percentage when *E. coli* in 11 samples is exposed to 16 and 30 µg/mL of tetracyclines were calculated and shown in Fig. 3. With respect to the control samples, the maximum percentage of elimination was 19% at 16 µg/mL and 76% at 30 µg/mL of tetracyclines. Moreover, 55% of detected *E. coli* in 11 samples demonstrated elimination percentages of ≥50% under the condition of 30 µg/mL of tetracyclines.

Further tests involving the three tetracycline-resistant genes (*tet* A, *tet* Q, and *tet* M) showed that the *tet* A and *tet* Q genes were not found, and they showed the presence of *tet* M with a concentration of (2.73 ± 0.261) × 10^7^ copies/mL, indicating that the antibiotics in the urine may motivate antibiotic resistance. However, antibiotic-resistant bacteria may also come from the environment, and their pathway remains unclear.

### 3.3 Changes in antibiotics and antibiotic-resistant bacteria and genes in stored source-separated urine

Urine-diverting toilets collect urine over time, and the samples are temporarily stored for weeks or months before further treatment or final utilization. We place the collected urine in storage for 30 days in an ambient environment to determine the effect of aging on the micropollutant changes (Table 7). The pH was 9.1–9.17 with an average of (9.14 ± 0.02) and the concentration of ammonia ranged between 4631.6–5389.8 mg/L with an average of (4951.4 ± 167) mg/L after 30 days of storage in the mixed samples. Three antibiotics remained detectable after 30 days of storage; fleroxacin decreased from 9.05 ng/mL to 0.344 ng/mL, enrofloxacin decreased from 11.44 ng/mL to 0.367 ng/mL, and pefloxacin decreased from 6.36 ng/mL to 4.66 ng/mL with a removal percentage of 96%, 97%, and 27%, respectively. Sulfamethoxazole, norfloxacin, ofloxacin, oxytetracycline, chlortetracycline, and doxycycline were below the detection limit after 5 days of storage. After 10 days of storage, sparfloxacin was reduced to under the detection limit from an initial concentration of 26.49 ng/mL. After 15 days of storage, the concentrations of orbifloxacin, sarafloxacin, and lomefloxacin became undetectable in the samples. The concentrations of danofloxacin and ciprofloxacin decreased from 64.42 ng/mL to 1.14 ng/mL and from 85.52 ng/mL to 2.25 ng/mL, respectively, after 20 days of storage but were reduced to under the detection limit after 25 days of storage. Difloxacin and tetracycline were undetected after 30 days of storage, and their last concentrations registered at 0.211 and 0.193 ng/mL, respectively, after 25 days of storage.

**Table 7:** Changes in antibiotic concentration during storage.

Antibiotic Group	Antibiotics	days						
		0	5	10	15	20	25	30
		ng/mL						
Sulfonamides	Sulfamethoxazole	2.54	ND	ND	ND	ND	ND	ND
Fluoroquinolones	Norfloxacin	89.9	ND	ND	ND	ND	ND	ND
	Fleroxacin	9.05	2.92	1.41	1.25	0.69	0.638	0.344
	Sparfloxacin	26.49	6.5	ND	ND	ND	ND	ND
	Orbifloxacin	4.97	2.72	1.41	ND	ND	ND	ND
	Enrofloxacin	11.44	2.44	1.17	1.1	0.695	0.366	0.367
	Dafloxacin	62.42	1.68	1.78	1.17	1.14	ND	ND
	Pefloxacin	6.36	4.03	3.445	2.84	1.753	4.062	4.66
	Difloxacin	10.65	1.74	0.72	0.435	0.384	0.211	ND
	Ciprofloxacin	85.52	4.87	2.07	1.995	2.25	ND	ND
	Sarafloxacin	94.41	3.96	1.44	ND	ND	ND	ND
	Lomefloxacin	68.95	1.92	0.96	ND	ND	ND	ND
	Ofloxacin	12.16	ND	ND	ND	ND	ND	ND
Tetracyclines	Tetracycline	2.69	0.656	0.39	0.254	0.322	0.193	ND
	Oxytetracycline	2.09	ND	ND	ND	ND	ND	ND
	Chlortetracycline	6.52	ND	ND	ND	ND	ND	ND
	Doxycycline	7.69	ND	ND	ND	ND	ND	ND

ND: not detected.

Drugs have the following possible fates in the environment: (1) biodegradable drugs may be mineralized into carbon dioxide and water, (2) drugs may undergo specific metabolic processes or be partially degraded, or (3) drugs may be persistent (Frade et al., 2014). In this study, urine samples remained untreated during storage, and hydrolysis was the main chemical process that could occur. Hydrolyzed urine may promote a biological and chemical process for antibiotic degradation. However, Jaatinen et al.(2016) obtained 1%–99% antibiotic removal with or without amendments throughout a urine storage period of 6 months and showed that bacterial activity and urea hydrolysis slightly influence the antibiotic removal efficiency. Thus, abiotic self-degradation may be the primary cause of antibiotic removal. Furthermore, risks associated with antibiotics may reduce when biodegradation and sorption occur in the soil after urine fertilization (Winker et al., 2008). However, this finding requires further evaluation because more than 100 antibiotics have been detected.

**Fig. 4:**
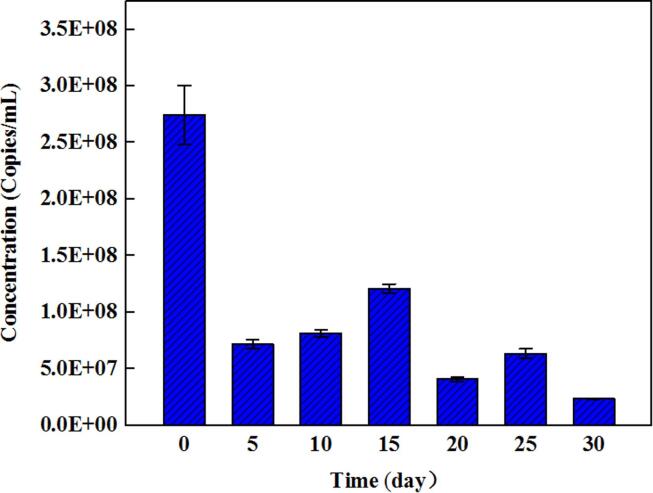
Concentration of *tet* M genes during storage.

The cell density of samples that are resistant to 30 µg/mL of tetracycline decreased from 120,000 UFC/100 mL to 5000 UFC/100 mL after 30 h of storage, which equals a reduction of 1.38 log, and decreased to be undetectable at 36 h. The concentration range of *tet* M was (2.73–0.261) × 10^8^ to (2.35 ± 0.0463) × 10^7^ copies/mL with a reduction of less than 1 log after 30 days of storage (Fig. 4), and the concentration remained high even after storage. A previous study demonstrated that ampicillin- and tetracycline-resistant genes carried by extracellular DNA released into aged urine demonstrate a low potential to spread antibiotic-resistant genes to bacteria when released to the environment (Goetsch et al., 2020). Meanwhile, a study demonstrated that the ARG in manure fertilized soil would transfer into the non-manured soils, and ARGs abundance below 1.62 × 10^9^ copies/g was suggested to be the typical level of non-manured soil. In regards to the *tet* M concentration found in the urine in this study, it seems that the concentration is safe to soil (Pu et al.,2020). However, this is only one strain, thus, the ecological risk should not be ignored and the gene transfer risk must be considered because the concentration remained high during storage. Overall, statistical changes in terms of log reduction were absent in antibiotic-resistant genes after 30 days of storage.

## 4 Conclusion

Antibiotics and antibiotic-resistant bacteria and genes were investigated in this study to further understand the risks of antibiotic-related issues in terms of urine utilization. The variation in antibiotics and representative antibiotic-resistant bacteria and genes during the temporary storage of urine was also investigated. Results showed that 18 antibiotics belonging to three groups (84% fluoroquinolones, 14% tetracyclines, and 2% sulfonamides) were detected in fresh source-separated urine at different concentrations and frequencies. Tetracycline-resistant *E. coli* was detected at a frequency of 55% with maximum cell densities of (200,000 ± 5000) CFU/100 mL, and *tet* M was detected with a frequency of 36% in the samples. Furthermore, during 30 days of storage, aging urine showed significant elimination of antibiotics and antibiotic-resistant bacteria but only a slight reduction in antibiotic-resistant genes. Therefore, such kinds of pollutants should be emphasized during urine treatment for the purpose of utilization because urine may act as a transport medium of antibiotics, antibiotic-resistant bacteria and genes.

## CRediT authorship contribution statement

**Xiaoqin Zhou:** Conceptualization, Writing - review & editing. **Gabriela Jacqueline Perez Cuasquer:** Investigation, Writing - original draft. **Zifu Li:** Supervision. **Heinz Peter Mang:** Supervision. **Yaping Lv:** Methodology.

## Declaration of Competing Interest

The authors declare that they have no known competing financial interests or personal relationships that could have appeared to influence the work reported in this paper.
