# Supplementary material for: Occurrence of typical antibiotics, representative antibiotic-resistant bacteria, and genes in fresh and stored source-separated human urine
Source: Environ Int. 2021 Jan;146:106280. doi: 10.1016/j.envint.2020.106280 (PMC7786438; doi:10.1016/j.envint.2020.106280)
Supplement: Supplementary Data 1 [file mmc1.docx]

**Supplementary information**

***Standards and stock solutions***

Reference substances of the highest available purity (purity>90%) were provided by China Institute of Veterinary Drug Con-trol (Beijing, China) and Sigma–Aldrich (St. Louis, MO, USA).The thirty antibiotics at highest available purity (>95%) were provided by the Institute of Quality Standard and Testing Technology for Agro-Products of Chinese Academy of Agricultural Sciences. Individual stock solutions (1000 mgL^−1^) were prepared by dissolving the compounds with acetonitrile and were then stored at −20◦C in darkness for a maximum of 3 months.

***Reagents and solvents***

The reagents used in the analysis were listed in Table S1. McIlvaine buffer was prepared by mixing citric acid solution and Na_2_HPO_4_ solution at a ratio of 8:5, and with 0.3% EDTA addition, thereafter adjusting the pH to 4. The citric acid solution was prepared by 8.4 g of citric acid with 400 mL pure water, while Na_2_HPO_4_ solution was 7.1 g of Na_2_HPO_4_ with 250 mL pure water.

**Table S1 Reagents used in the antibiotic’s analysis**

| **No.** | **Name** | **Manufacturers** | **Notes** |
| --- | --- | --- | --- |
| 1 | Acetonitrile (ACN) | CNW Technologies GmbH (Düsseldorf, Germany) | HPLC grade |
| 2 | methanol (MeOH) |  | HPLC grade |
| 3 | Formic acid | Sigma (Sigma-Aldrich, St. Louis, USA) | high quality grade |
| 4 | Citric acid monohydrate (C_6_H_8_O_7_·H_2_O) | J. T. Baker (Phillipsburg, NJ) | Analytical grade |
| 5 | disodium hydrogen phosphate (Na_2_HPO_4_) |  | Analytical grade |
| 6 | ethylenediaminetetraacetic acid disodium salt dehydrate (Na_2_EDTA·2H_2_O) |  | Analytical grade |
| 7 | anhydrous sodium sulfate (NaSO_4_), | Materials Co. (Shanghai, China). |  |
| 8 | sodium chloride (NaCl) |  |  |
| 9 | primary secondary amine (PSA) |  |  |
| 10 | octadecylsilane (C18) |  |  |
| 11 | SPE Oasis HLB cartridges (3 ml, 60 mg) | Waters (Milford, MA, USA) |  |

***Sample extraction***

2 mL of urine sample was transferred into 50 ml polypropylene centrifuge tubes, then adding in 10 mL of EDTA-McIlvaine buffer and 6 mL acetonitrile solution for vortex mixing. Then placed into an ultrasonic bath for 20 min, followed by centrifuged at 15000 rpm for 10 min and the supernatant was decanted into a 50 ml centrifuge(supernatant1); The supernatant from the supernatant1 was subjected to liquid–liquid adding solid NaCl (1 g) and Na_2_SO_4_ (4 g). The tube was immediately sealed the tube and then shaken vigorously by hand for 1 min. The tube was centrifuged at 10000 rpm for 5 min and the supernatant was pipetted into a 50 ml（supernatant2）. Followed by, 4ml methanol was added into the supernatant1 for 1min vortex mixing, then centrifugation at 10000 rpm for 5 min to extracted supernatant again(supernatant2). 6 mL Mixture of the two supernatants together then transferred to 10 ml polypropylene centrifuge tube. Solution containing 900 mgNa_2_SO_4_ and 50 mg of PSA sorbent and 150 mg C18 was added in for purification. The mixture was vortexed for 1 min until the sorbent material was thoroughly dispersed and centrifuged again for5 min at 5000 rpm. After centrifugation, the supernatant was dried with nitrogen 5mL，with methanol− 0.1% formic acid aqueous solution (6:4, V/V) dissolved vortex mixing 1min, centrifuged for 10000 r/min 2 min, through a 0.22 m nylon membrane filter (Whatman, U.K.) prior to LC–MS/MS analysis.
